# Actions of Probiotics on Trinitrobenzenesulfonic Acid-Induced Colitis in Rats

**DOI:** 10.1155/2015/528523

**Published:** 2015-10-13

**Authors:** Takahiko Shiina, Takeshi Shima, Kiyotada Naitou, Hiroyuki Nakamori, Yuuki Sano, Kazuhiro Horii, Masaki Shimakawa, Hiroshi Ohno, Yasutake Shimizu

**Affiliations:** ^1^Department of Basic Veterinary Science, Laboratory of Physiology, The United Graduate School of Veterinary Sciences, Gifu University, 1-1 Yanagido, Gifu 501-1193, Japan; ^2^Biofermin Kobe Research Institute, Biofermin Pharmaceutical Co. Ltd., 7-3-4 Ibukidai-Higashimachi, Nishi-ku, Kobe 651-2242, Japan

## Abstract

We investigated the actions of probiotics, *Streptococcus faecalis* 129 BIO 3B (SF3B), in a trinitrobenzenesulfonic acid- (TNBS-) induced colitis model in rats. After TNBS was administered into the colons of rats for induction of colitis, the rats were divided into two groups: one group was given a control diet and the other group was given a diet containing SF3B for 14 days. There were no apparent differences in body weight, diarrhea period, macroscopic colitis score, and colonic weight/length ratio between the control group and SF3B group, suggesting that induction of colitis was not prevented by SF3B. Next, we investigated whether SF3B-containing diet intake affects the restoration of enteric neurotransmissions being damaged during induction of colitis by TNBS using isolated colonic preparations. Recovery of the nitrergic component was greater in the SF3B group than in the control group. A compensatory appearance of nontachykininergic and noncholinergic excitatory components was less in the SF3B group than in the control group. In conclusion, the present study suggests that SF3B-containing diet intake can partially prevent disruptions of enteric neurotransmissions induced after onset of TNBS-induced colitis, suggesting that SF3B has therapeutic potential.

## 1. Introduction

Inflammatory bowel disease (IBD) is a group of chronic, incurable inflammatory disorders of the gastrointestinal tract, including Crohn's disease and ulcerative colitis [1, 2]. IBD has become almost a global disease affecting people of almost all ages including the pediatric population [3, 4]. A convenient approach to study the pathogenesis of human IBD is to use animal models of IBD [4, 5]. Various animal models have been established to study IBD including chemical-induced colitis models such as trinitrobenzenesulfonic acid (TNBS), dextran sodium sulphate (DSS), and oxazolone-induced colitis models [1, 4].

Intracolonic application of TNBS induces colonic inflammation characterized by increased leukocyte infiltration, edema, and ulceration [4]. In addition, TNBS-induced colitis leads to alterations in enteric neuronal transmission regulating gastrointestinal motility, which is commonly found in IBD [6–10]. For instance, we previously demonstrated by using a TNBS-induced colitis model that the colonic inflammation causes indiscriminate damage to enteric neurons and that noncholinergic nontachykininergic excitatory neural components appear during restoration of inflammation probably as a result of a compensatory neurogenesis [11]. TNBS-induced colitis model animals have been widely used for identifying anti-inflammatory components such as plant extracts [12], seed oils [13], and probiotics [2, 14].

Probiotics are organisms that provide a desired and beneficial effect on human health. The probiotic* Streptococcus faecalis* 129 BIO 3B (SF3B: strain currently classified as* Enterococcus faecium*), which is a normal bowel commensal and lactic acid bacterium, has been reported to have various actions such as intestinal regulation [15, 16]. SF3B attenuated abdominal pain in an experimental rat model of TNBS-induced visceral hypersensitivity [16], suggesting that SF3B might affect the pathogenesis of TNBS-induced dysfunctions including colitis. In addition, growing evidence for a role of intestinal microflora in the development of IBD has led to studies on the therapeutic potential of modifying the composition of intestinal microbiota using probiotics [14]. In accordance with this, Yoshimatsu et al. recently reported that probiotics might be effective for maintaining clinical remission in patients with quiescent ulcerative colitis [17]. Hence, in the present study, we studied actions of probiotics on TNBS-induced colitis with focus on the alteration/recovery of neural components of the enteric nervous system that regulates intestinal motility.

## 2. Materials and Methods

### 2.1. Animals

Male Wistar rats (8–11 weeks of age, 200–300 g in weight) were obtained from Japan SLC (Shizuoka, Japan). They were maintained in plastic cages at 24 ± 2°C with a 12:12-hour light-dark cycle (light on at 08:00–20:00 h) and given free access to food and water. The Animal Care and Use Committee of Gifu University approved the animal experiments (experiment numbers: 12114, 13086, and 14108).

### 2.2. Preparation of Bacterial Powder and Bacterial Powder-Containing Diet

Dry powder consisting of SF3B (5 × 10^11^ cfu/g) isolated from human feces and a vehicle composed mainly of dextrin were used. This powder was supplied by Biofermin Pharmaceutical Co. Ltd. (Kobe, Japan). The compositions of diets used in this experiment are shown in Table 1.

### 2.3. Induction of Colitis and Feeding Program

After overnight fasting, animals were anesthetized with isoflurane. Then, they were given 1 mg of TNBS dissolved in 0.8 mL of 40% ethanol (v/v) by means of a silicon catheter inserted 7 cm through the anus. The animals were maintained in a head-down position for about 1 min to prevent leakage of the intracolonic solution. Animals were divided into two groups: one group was given a control diet (CONT group) and the other group was given a diet containing SF3B (SF3B group). The animals were maintained in their plastic cages and used at 7 days or 14 days after receiving TNBS. Macroscopic colitis score was calculated according to the criteria shown in Table 2.

### 2.4. Tissue Preparation

Animals were anesthetized by isoflurane and exsanguinated via the carotid artery. The abdominal cavity was opened immediately, and a 3-4 cm long segment of the distal colon (2 cm from the anus) was dissected out and immersed in modified Tyrode's solution (see below) at room temperature. The intraluminal contents were flushed using a small cannula filled with modified Tyrode's solution.

### 2.5. Mechanical Recordings

A segment of the distal colon of 3-4 cm length was mounted in a Magnus tube (20 mL in capacity) filled with modified Tyrode's solution (pH 7.4). The solution was continuously bubbled with 95% O_2_ + 5% CO_2_ gas mixture and maintained at 37°C. The distal end of each segment was tied to organ holders and the proximal end was secured with a silk thread to an isometric force transducer. The preparation was stimulated electrically by means of two platinum electrodes, one of which was placed in the lumen of the preparation and the other in the bathing solution (coaxial stimulation). Supramaximal rectangular pulses of 80 V in intensity and 0.5 msec in duration were delivered by using an electrical stimulator (model SEN-3301, Nihon Kohden, Tokyo, Japan) with frequency spectra of 20 Hz for 1 sec. Longitudinal contractile activity was recorded isometrically with a force transducer (T7-8-240, Orientec, Tokyo, Japan). An initial tension of 1.0 g was applied to the colonic preparations, which were subsequently allowed to equilibrate for 30 min. At the end of this period, the tension created by the segment was considered as the resting tension and no further mechanical adjustment was made during experimentation. Isometric responses were filtered and amplified by an amplifier (NEC, AS1202, Tokyo, Japan) and recorded using a PowerLab system (AD Instruments, Bella Vista, NSW, Australia).

### 2.6. Solutions and Drugs

During experiments for recording mechanical responses, tissues were maintained in modified Tyrode's solution consisting of (in mM) NaCl 136.9, KCl 2.68, CaCl_2_ 1.80, MgCl_2_ 1.0, NaH_2_PO_4_ 0.41, NaHCO_3_ 11.90, and glucose 5.55. Tetrodotoxin was used as a blocker of voltage-dependent sodium channels on neurons. Atropine was used to block muscarinic acetylcholine receptors on smooth muscle cells. N-Acetyl-L-tryptophan 3,5-bis (trifluoromethyl) benzyl ester (L-732,138) and (S)-N-methyl-N[4-(4-acetylamino-4-phenylpiperidino)-2-(3,4-dichlorophenyl) butyl] benzamide (SR48968) were used to block tachykinin NK_1_ and tachykinin NK_2_ receptors, respectively. N*ω*-Nitro-L-arginine methyl ester hydrochloride (L-NAME) was used as a nitric oxide (NO) synthase (NOS) inhibitor. L-732,138 and L-NAME were obtained from Sigma (St. Louis, MO, USA). Atropine sulfate salt monohydrate, TNBS, and tetrodotoxin were obtained from Wako (Osaka, Japan). SR48968 was a gift from Sanofi-Synthelabo (Montpellier, France). L-732,138 and SR48968 were dissolved in ethyl acetate and ethanol, respectively. The vehicles (ethanol and ethyl acetate) for the drugs alone had no effect on basal tone. Other drugs were dissolved in distilled water. The drug concentrations given in the test were final concentrations in the bath solution.

### 2.7. RNA Isolation and Reverse Transcription-Polymerase Chain Reaction (RT-PCR)

The expression of mRNA was assessed by RT-PCR. Total cellular RNA was extracted from tissue homogenates of the rat colon using TRIzol reagent (Thermo Fisher Scientific Inc., Waltham, MA, USA). First-strand cDNA was synthesized from 2 *μ*g of total RNA by using SuperScript III Reverse Transcriptase (Fisher Scientific Inc.) and Random Primers (Fisher Scientific Inc.). The absence of PCR-amplified DNA fragments in the samples without reverse transcription indicated the isolation of RNA free from genomic DNA contamination. The PCR was performed with platinum Taq DNA polymerase (Fisher Scientific Inc.). The primer sets were as follows: inducible NOS (iNOS) sense 5′-TAG AAA CAA CAG GAA CCT AC-3′ and antisense 5′-AAC ATC TCC TGG TGG AAC A-3′ (predicted size = 925 bp); neuronal NOS (nNOS) sense 5′-CCG GAA TTC GAA TAC CAG CCT GAT CCA TGG AA-3′ and antisense 5′-CCG AAT TCC TCC AGG AGG GTG TCC ACC GCA TG-3′ (predicted size = 617 bp); c-Myc sense 5′-AAG AGA ATT TCT ATC ACC AG-3′ and antisense 5′-TTG ATG GGG ATG ACC CTG AC-3′ (predicted size = 771 bp); CyclinD1 sense 5′-AGG AGC AGA AGT GCG AAG AG-3′ and antisense 5′-TAG CAG GAG AGG AAG TTG TT-3′ (predicted size = 475 bp); leucine-rich repeat containing G protein-coupled receptor 5 (Lgr5) sense 5′-GCG GGG CTG CCC ATC ATA CT-3′ and antisense 5′-TTG CTG TGG AAT CCT AGT TC-3′ (predicted size = 708 bp); hairy and enhancer of split-1 (Hes1) sense 5′-AGA AAA ATT CCT CGT CCC CG-3′ and antisense 5′-GGA AGC CGC CAA AAA CCT TG-3′ (predicted size = 642 bp); atonal protein homolog 1 (Atoh1) sense 5′-ACC ACC ATC GCC ATC CCC AG-3′ and antisense 5′-CGC CTG CTC CTC CTT CAT AA-3′ (predicted size = 687 bp); Notch1 sense 5′-GAG GTG CGA AGT GGC CAA CG-3′ and antisense 5′-AGT CCA GCC ATT GAC ACA CA-3′ (predicted size = 880 bp); and *β*-actin sense 5′-TGA CCC TGA AGT ACC CCA TTG-3′ and antisense 5′-TCA GGA TCT TCA TGA GGT AG-3′ (predicted size = 387 bp). All primers were purchased from Eurofins Genomics (Tokyo, Japan). Amplifications were performed by 25 or 30 cycles. The reaction products were electrophoresed on 1.5% agarose gels and stained with ethidium bromide (0.4 *μ*g/mL). The gels were exposed to UV light with a UV transilluminator (UVP Laboratory Products, Upland, CA, USA) and photographed. Densitometry analyses of the results were performed with ImageJ software. Relative values of mRNA expression were calculated by taking the value of the respective *β*-actin mRNA as unity (1.0).

### 2.8. Macroscopic Assessment of Colitis

The isolated colon segments were examined visually, and their damage was scored. Macroscopic colitis scores were assigned values of 0–6 as shown in Table 2. In addition, colonic weight/length ratio was calculated as a parameter for evaluating inflammation damage, since colonic inflammation can result in an increase in weight of the colon and decrease in length of the colon [18].

### 2.9. Data Analysis

Data are presented as means ± standard deviation (S.D.). *n* indicates the number of experiments performed using different tissue preparations from different animals. The significance of differences between mean values was determined by one-way or two-way analysis of variance followed by the Turkey-Kramer test for comparison of multiple groups or by Student's *t*-test or Mann-Whitney *U* test for comparison of two groups. A *P* value less than 0.05 denotes the presence of a statistically significant difference.

## 3. Results

### 3.1. Effects of SF3B-Containing Diet Intake on TNBS-Induced Colitis in Rats

**Figure 1:**
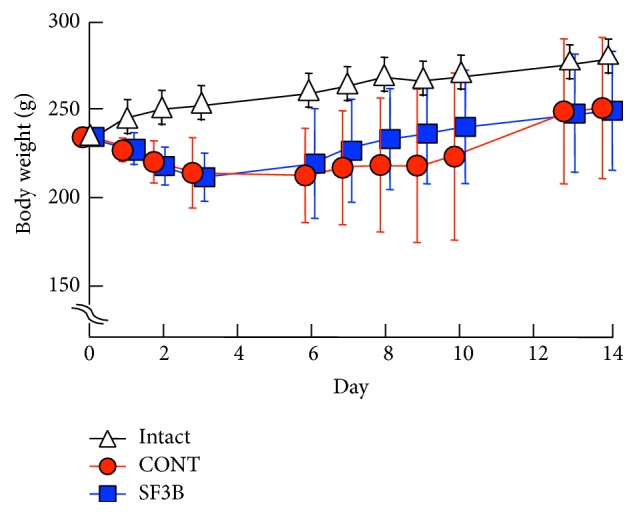
Body weight changes in TNBS-treated rats taking the control diet (CONT group; *n* = 8) or SF3B-containing diet (SF3B group; *n* = 10) and intact rats (*n* = 4). At day 0, TNBS was injected into the distal colon. Each value represents the mean ± S.D.

**Figure 2:**
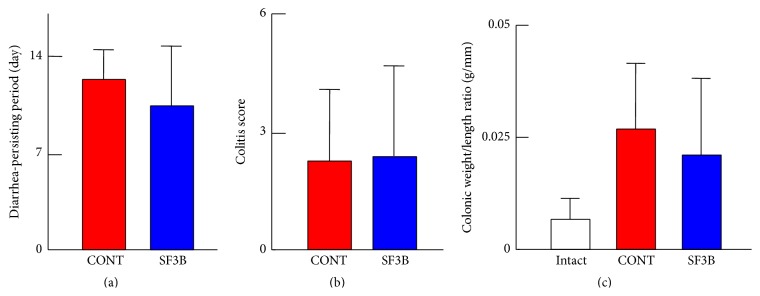
Effects of SF3B-containing diet intake on TNBS-induced colitis in rats. (a) Summary graphs showing diarrhea-persisting periods (days) in the CONT group (*n* = 9) and SF3B group (*n* = 9). (b) Summary graphs showing macroscopic colitis scores in the CONT group (*n* = 7) and SF3B group (*n* = 10) at 14 days after TNBS treatment (see Table 2). (c) Summary graphs showing colonic weight/length ratios in the CONT group (*n* = 7) and SF3B group (*n* = 10) at 14 days after TNBS treatment and in the intact group (*n* = 4). Each bar represents the mean ± S.D.

TNBS was injected into the distal colon of each rat to induce colitis, and then a control diet or a diet containing SF3B was given for 14 days. Body weights in both the CONT group and SF3B group decreased within the initial 3 days after treatment with TNBS but recovered gradually as days passed (Figure 1). There was no significant difference in body weight changes between the groups. In contrast, the weight of intact animals increased during the same period. Diarrhea occurred 1–3 days after TNBS treatment and persisted for about 10–12 days in both groups (Figure 2(a)). Macroscopic observation showed that treatment with TNBS induced visible inflammation in the colon, as indicated by the macroscopic colitis score (Table 2): close to 0 in intact rats versus about 2-3 in the CONT group and SF3B group at 14 days after TNBS treatment (Figure 2(b)). Colonic weight/length ratios in the CONT group and SF3B group at 14 days after TNBS treatment were higher than the ratio in the intact group, while there was no significant difference between the CONT group and SF3B group (Figure 2(c)).

### 3.2. Effects of SF3B-Containing Diet Intake on Inflammation-Induced Changes in Nitrergic Responses in Colonic Motor Functions

**Figure 3:**
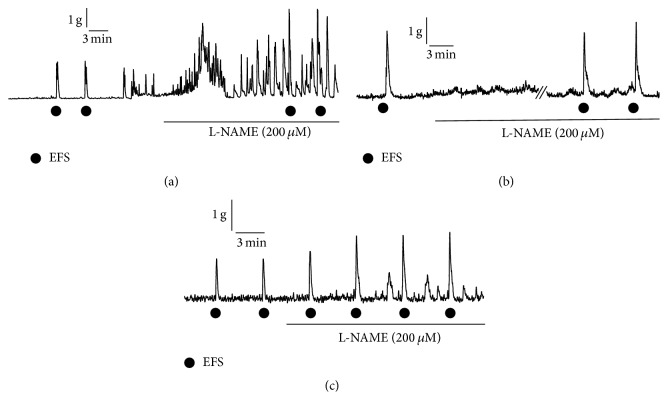
Effects of a NOS inhibitor on spontaneous contractions and electrical field stimulation- (EFS-) induced contractions in isolated colonic preparations from rats. Representative tracings of mechanical responses in the absence and presence of a NOS inhibitor, L-NAME (200 *μ*M), in an intact rat (a), a TNBS-treated rat with the control diet at 14 days after TNBS treatment (b), and a TNBS-treated rat with the SF3B-containing diet at 14 days after TNBS treatment (c) are shown. Circles indicate the points of EFS.

At 14 days after TNBS treatment, we investigated functions of enteric neurons in motility of the inflamed colon using an* in vitro* system. In segments of the distal colon from the intact group, spontaneous contractions were observed (Figure 3(a)). Application of EFS induced contractile responses in the rat colon (Figure 3(a)). The EFS-induced contractile response was abolished by application of tetrodotoxin (1 *μ*M), a blocker of voltage-dependent sodium channels on neurons (data not shown). To determine whether NO influences mechanical responses in the rat colon, L-NAME (200 *μ*M), a NOS inhibitor, was applied. In all of the preparations from the intact group (*n* = 7), as shown in Figure 3(a), L-NAME application increased the frequency and amplitude of spontaneous contractions, suggesting that the colonic contractile activity is subject to tonic nitrergic inhibitory control. In agreement with this, amplitude of the EFS-induced contraction was increased after application of L-NAME (Figure 3(a)). In contrast, 36.4% of the preparations from the CONT group (*n* = 11) were resistant to L-NAME: spontaneous contractions and EFS-induced contractions were not enhanced even in the presence of L-NAME (Figure 3(b)), indicating reduction of the tonic nitrergic inhibition. However, all of the preparations from the SF3B group (*n* = 9) were sensitive to L-NAME application as were the preparations from the intact group (Figure 3(c)).

### 3.3. Effects of SF3B-Containing Diet Intake on Inflammation-Induced Changes in Excitatory Responses in Colonic Motor Functions

**Figure 4:**
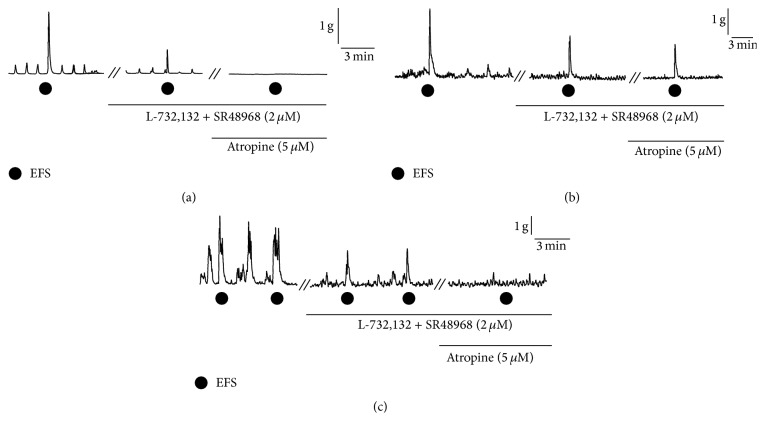
Effects of tachykinergic and cholinergic inhibitors on EFS-induced contractions in isolated colonic preparations from rats. Representative tracings of mechanical responses in the absence and presence of blockers for tachykinin NK_1_ receptors and tachykinin NK_2_ receptors (L-732,132 and SR48968, 2 *μ*M, resp.) and atropine, a blocker for muscarinic cholinoceptors (5 *μ*M), in an intact rat (a), a TNBS-treated rat with the control diet at 14 days after TNBS treatment (b), and a TNBS-treated rat with the SF3B-containing diet at 14 days after TNBS treatment (c), are shown. Circles indicate the points of EFS. To simplify the interpretation of results of pharmacological experiments, blockers were applied under the condition in which the inhibitory components were eliminated by L-NAME (200 *μ*M).

Next, we examined the effect of SF3B-containing diet on neuronal components regulating EFS-induced contractile responses in the inflamed colon at 14 days after TNBS treatment. To simplify the interpretation of results of pharmacological experiments, blockers were applied under the condition in which the inhibitory components were eliminated by L-NAME (200 *μ*M). In preparations of the intact group (*n* = 7), as shown in Figure 4(a), combined application of blockers for tachykinin NK_1_ receptors and tachykinin NK_2_ receptors (L-732,132 and SR48968, 2 *μ*M, resp.) significantly diminished the EFS-evoked contractile responses (Figure 4(a)). The contraction being resistant to tachykininergic antagonists was blocked by the cumulative application of atropine (5 *μ*M), a blocker for muscarinic cholinoceptors (Figure 4(a)). In contrast, in the majority of preparations from the CONT group (*n* = 11), EFS-induced contractile response was not completely blocked by combined application of tachykininergic and cholinergic antagonists (Figure 4(b)). The noncholinergic/nontachykininergic response was abolished by tetrodotoxin (1 *μ*M), an inhibitor of neurogenic responses (data not shown). In contrast, in half of the preparations from the SF3B group (*n* = 9), combined application of tachykininergic and cholinergic antagonists almost totally abolished EFS-induced contraction in a manner similar to that in the intact group (Figure 4(c)).

### 3.4. Effects of SF3B-Containing Diet Intake on Expression of mRNAs of Inflammation-Related and Tissue Regeneration-Related Genes and an Enteric Neuron-Expressing Gene in the Inflamed Colon

**Figure 5:**
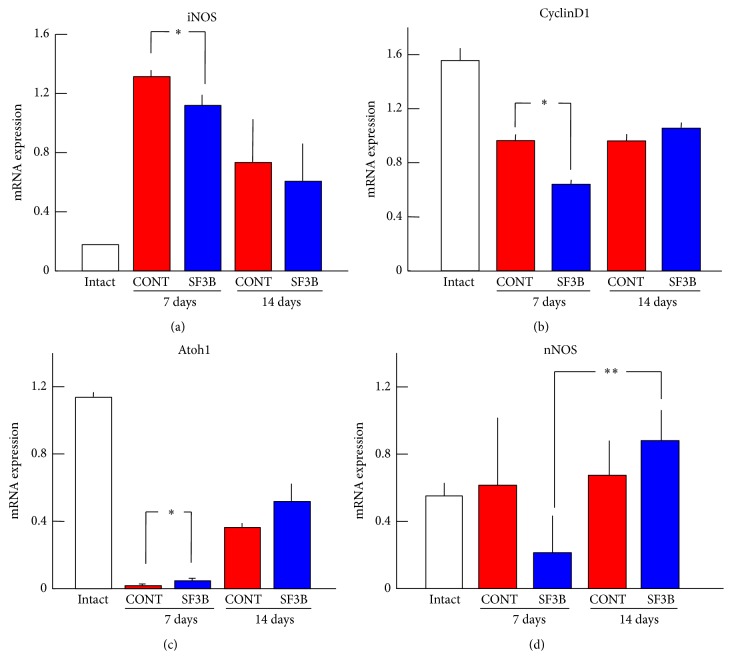
Effects of SF3B-containing diet intake on expression of mRNAs of inflammation-related and tissue regeneration-related genes in the inflamed colon. Expression levels of iNOS (a), CyclinD1 (b), Atoh1 (c), and nNOS (d) genes in colons of the CONT group (*n* = 5) and SF3B group (*n* = 5) at 7 or 14 days after TNBS treatment and of the intact group (*n* = 3) were analyzed by RT-PCR. Summary graphs showing relative values of mRNA expression calculated by taking the value of the respective *β*-actin mRNA as unity (1.0). Each bar represents the mean ± S.D. ^*∗*^ *P* < 0.05 and ^*∗∗*^ *P* < 0.01, comparison between the CONT group and SF3B group at the same day after TNBS treatment or between the SF3B groups at different day after TNBS treatment.

Expression of iNOS, Atoh1, CyclinD1, c-Myc, Lgr5, Hes-1, Notch1, and nNOS genes in the inflamed colon was analyzed by RT-PCR. After treatment with TNBS, the expression of iNOS mRNA was increased, while the expressions of other inflammation-related and tissue regeneration-related gene mRNAs were decreased (Figures 5(a), 5(b), and 5(c)). Expression levels of iNOS and CyclinD1 mRNAs in the SF3B group were lower than those in the CONT group at 7 days after treatment with TNBS (Figures 5(a) and 5(b)). On the other hand, expression level of Atoh1 mRNA in the SF3B group was higher than that in the CONT group at 7 days after TNBS application (Figure 5(c)). In addition, expression level of nNOS mRNA in the SF3B group at 14 days after treatment with TNBS was higher than that in the SF3B group at 7 days after TNBS application (Figure 5(d)). There was no significant difference in expression levels of other genes between the SF3B group and CONT group.

## 4. Discussion

In the present study, we tried to determine whether SF3B has beneficial actions on IBD, especially the recovery phase of IBD, by using a TNBS-induced colitis model in rats. We found no apparent differences in body weight, diarrhea period, macroscopic colitis score, and colonic weight/length ratio between the control diet group and SF3B-containing diet group. On the basis of these results, induction and progression of colitis itself did not seem to be prevented even by taking SF3B-containing diet at least in this experimental condition. However, a remarkable finding of our study is that enteric neural components, which are impaired by an acute inflammatory process, are normally restored by ingestion of an SF3B-containing diet. Considering that alterations in enteric neural components being promoted following transient intestinal inflammation, as manifested in the CONT group in this study, can be a cause of abnormal intestinal motility [11, 19–21], the action of SF3B to bring about normal restoration would be beneficial for treatment of IBD.

Disruption of enteric neurotransmissions is one of the principle causes of colitis-induced abnormal intestinal motility [7–11, 22, 23]. In the present study, we investigated whether ingestion of an SF3B-containing diet affects restoration of nitrergic, tachykininergic, and cholinergic neurotransmissions being damaged during induction of colitis by TNBS. Recovery of the nitrergic component as assessed by increases in frequency and amplitude of spontaneous contractions after application of L-NAME was greater in the SF3B group than in the CONT group. This suggests that ingestion of SF3B accelerates restoration of the nitrergic component. In accordance with this, the expression level of nNOS gene at 14 days after treatment with TNBS was higher than that at 7 days after treatment with TNBS in the SF3B group but not in the CONT group. Since the inhibitory nitrergic component of the enteric nervous system plays an important role in the regulation of intestinal motility, the effect of SF3B would be accessible to avoid persistent alteration of motility patterns commonly observed after the resolution of intestinal inflammation [19, 21]. In addition, appearance of nontachykininergic and noncholinergic excitatory components was less in the SF3B group than in the CONT group. The appearance of unusual neural components can be considered to be a compensatory mechanism to maintain intestinal motility [11]. However, excessive compensation might not always be beneficial for normal functions. Thus, less expression of the nontachykininergic and noncholinergic excitatory components can be appreciated as a valuable action of SF3B.

IBD is an important etiologic factor in the development of colorectal cancer [24]. It has been pointed out that several molecular alterations associated with IBD are related to colorectal cancer. For instance, activation of CyclinD1-mediated cell proliferation and high activity of iNOS might contribute to colon oncogenesis [20, 25]. Interestingly, SF3B-containing diet intake reduced the expression of iNOS and CyclinD1 mRNA compared to that in rats fed the control diet. It is therefore expected that the risk for IBD-associated colorectal cancer would be alleviated by the action of SF3B.

Although probiotics have been reported to exert various actions in humans and animals, the mechanisms of their actions are not completely understood [14]. Probiotics can occupy the physical space and interact with intestinal tissue or release bioactive products/metabolites, resulting in the blocking of pathogenic bacteria, enhancement of barrier function, and alteration of the mucosal immune system [14]. In fact, it had been reported that ingestion of* Streptococcus faecalis* resulted in increasing bifidobacteria and enterococci of the intestinal flora in rats [26]. Moreover, probiotics can exert anti-inflammatory action by reducing the production of proinflammatory cytokines [14]. In accordance with this, SF3B-containing diet intake inhibited expression of a proinflammation marker gene iNOS. Considering that enteric neuroplasticity evoked by inflammation such as that in IBD might be caused by proinflammatory cytokines from activated immune cells [6, 7, 14], SF3B might prevent colitis-associated disruptions of enteric neurons by blocking activation of the inflammatory response at least partially.

In conclusion, the present study suggests that ingestion of an SF3B-containing diet can partially prevent disruptions of enteric neurotransmissions induced after onset of TNBS-induced colitis and inhibit the expression of genes involved in oncogenesis, suggesting that SF3B has therapeutic potential.

## Figures and Tables

**Table 1:** Compositions of experimental diets (%).

	Control diet	SF3B diet
CE-2^*∗*^	90	90
SF3B powder	—	10
Dextrin	10	—

^*∗*^CE-2: standard diet (CLEA Japan, Tokyo, Japan).

**Table 2:** Criteria for scoring of colitis by macroscopic assessment.

Feature graded	Grade	Description
Feces	0	Normal
	1	Loose

Mucosa damage	0	None
	1	Mild
	2	Moderate
	3	Severe

Adhesion	0	None
	1	Mild
	2	Severe
